# Identification and Characterization of Cannabidiol as an OX1R Antagonist by Computational and In Vitro Functional Validation

**DOI:** 10.3390/biom11081134

**Published:** 2021-08-01

**Authors:** Rosa Maria Vitale, Fabio Arturo Iannotti, Aniello Schiano Moriello, Lea Tunisi, Fabiana Piscitelli, Ranjev Savopoulos, Luigia Cristino, Luciano De Petrocellis, Pietro Amodeo, Roy Gray, Vincenzo Di Marzo

**Affiliations:** 1 Institute of Biomolecular Chemistry, National Research Council (ICB-CNR), Via Campi Flegrei 34, 80078 Pozzuoli, Italy; fabio.iannotti@icb.cnr.it (F.A.I.); fpiscitelli@icb.cnr.it (F.P.); luigia.cristino@icb.cnr.it (L.C.); luciano.depetrocellis@icb.cnr.it (L.D.); pamodeo@icb.cnr.it (P.A.); 2 Endocannabinoid Research Group (ERG), Institute of Biomolecular Chemistry, National Research Council (ICB-CNR), Via Campi Flegrei 34, 80078 Pozzuoli, Italy; aniello.schianomoriello@icb.cnr.it (A.S.M.); lea.tunisi@gmail.com (L.T.); 3 Epitech Group SpA, Saccolongo, 35100 Padova, Italy; 4 Department of Veterinary Medicine and Animal Productions, University of Naples Federico II, 80131 Naples, Italy; 5 GW Research Ltd., Sovereign House, Vision Park, Histon, Cambridge CB24 9BZ, UK; RSavopoulos@gwpharm.com (R.S.); RGray@gwpharm.com (R.G.); 6 Canada Excellence Research Chair on the Microbiome-Endocannabinoidome Axis in Metabolic Health (CERC-MEND), Université Laval, Quebec City, QC G1V 4G5, Canada

**Keywords:** orexin receptors, cannabidiol, molecular docking, molecular dynamics, calcium mobilization assay

## Abstract

The potential, multifaceted therapeutic profile of cannabidiol (CBD), a major constituent derived from the *Cannabis sativa* plant, covers a wide range of neurological and psychiatric disorders, ranging from anxiety to pediatric epilepsy and drug addiction. However, the molecular targets responsible for these effects have been only partially identified. In this view, the involvement of the orexin system, the key regulator in arousal and the sleep/wake cycle, and in motivation and reward processes, including drug addiction, prompted us to explore, using computational and experimental approaches, the possibility that CBD could act as a ligand of orexin receptors, orexin 1 receptor of type 1 (OX1R) and type 2 (OX2R). Ligand-binding assays showed that CBD is a selective ligand of OX1R in the low micromolar range (Ki 1.58 ± 0.2 μM) while in vitro functional assays, carried out by intracellular calcium imaging and mobilization assays, showed that CBD acts as an antagonist at this receptor. Finally, the putative binding mode of CBD has been inferred by molecular docking and molecular dynamics simulations and its selectivity toward the OX1R subtype rationalized at the molecular level. This study provides the first evidence that CBD acts as an OX1R antagonist, supporting its potential use in addictive disorders and/or body weight regulation.

## 1. Introduction

Cannabidiol (CBD), the main non-euphoric constituent of *Cannabis sativa,* exhibits a complex neuropharmacological profile, ranging from anxiolytic, anticonvulsive, and neuroprotective properties to analgesic and anti-inflammatory effects, all potentially useful for the treatment of several neurological and psychiatric disorders [1,2]. The molecular targets of CBD likely involved in its therapeutic effects have been recently reviewed [3,4] and include, among others, the transient potential receptor channels (TRPs) TRPV1, TRPA1, and TRPM8 [5], the G-protein coupled receptors GPR55 [6], serotonin 1A receptor [7], and opioid receptors [8]. Moreover, even though CBD has a low affinity for the cannabinoid receptors, recent evidence indicated that it may act as a cannabinoid receptor of type 1 (CB1R)-negative allosteric modulator in some circumstances [9,10,11]. More recently, CBD has been also proposed as a therapeutic tool for addictive disorders such as drug and alcohol abuse [12,13,14]. In this view, the recent findings that the orexin system is involved in drug addiction [15], prompted us to investigate the orexin receptors, OX1R and OX2R, as possible targets for CBD. Both receptors are coupled with a Gq protein and their activation induces cell responses mainly via calcium (Ca^2+^)- and diacylglycerol (DAG)-mediated pathways [16,17]. The endogenous ligands of these G-protein coupled receptors are two hypothalamic neuropeptides, orexin-A (OX-A) and orexin-B (OX-B), which regulate different physiological functions in mammals, such as sleep-wake cycles, energy metabolism, and feeding [18]. Both peptides arise from a common precursor, the prepro-orexin, a 130-amino-acid peptide. Orexin projections extend widely throughout the brain, innervating the neocortex, hippocampus, and forebrain structures implicated in the processing of arousal [19], wakefulness [20], feeding [21], emotion, and motivation [22]. Defects in the orexin system induce narcolepsy and catalepsy, whereas inhibitors of orexin receptors represent a promising therapy for the treatment of sleep disorders [23]. Orexin 1 and Orexin 2 receptors are differently distributed in the central nervous system (CNS), thus reflecting different biological profiles—while OX2R is mainly involved in sleep and arousal, OX1R is implicated in compulsive behaviour related to drug addiction and anxiety [24]. OX1R has a higher affinity for OX-A than OX-B, whereas OX2R binds both OX-A and OX-B with the same affinity [23]. In the present study, by performing binding experiments on both orexin receptors, we found that CBD binds OX1R selectively over OX2R. Then, by exploiting the recently-released X-ray structures of both orexin receptors [25,26], the putative binding modes of CBD to OX1R were investigated by molecular docking and molecular dynamics simulations. Computational data, in addition to providing a possible structural basis for the observed binding, also allowed a rationalization of CBD selectivity toward the OX1R subtype. Finally, functional studies based on the measurement of intracellular calcium imaging and mobilization in OX1R-transfected Chinese hamster ovary (CHO) cells allowed the characterization of CBD as an OX1R antagonist.

## 2. Materials and Methods

### 2.1. Radioligand Binding Assay on CHO Cells Expressing hOX1R

Radioligand binding assays were carried out at Eurofins Cerep (France). Cell membrane homogenates (20 µg protein) were incubated for 60 min at 22 °C with 0.1 nM [^125^I] orexin-A in the absence or presence of plant-derived CBD (GW Research Ltd., Cambridge, UK) in a buffer containing 25 mM 4-(2-hydroxyethyl)-1-piperazineethanesulfonic acid (HEPES)/NaOH (pH 7.4), 0.5 mM EDTA, 2.5 mM CaCl_2_, and 2.5 mM MgCl_2_. Nonspecific binding was determined in the presence of 1 µM SB 334867. Following incubation, the samples were filtered rapidly under vacuum through glass fiber filters (GF/B, Packard Shelton, CT, USA) presoaked with 0.3% polyethylenimine (PEI) and rinsed several times with an ice-cold buffer containing 50 mM Tris-HCl and 150 mM NaCl using a 96-sample cell harvester (Unifilter, Packard, Shelton, CT, USA). The filers were dried then counted for radioactivity in a scintillation counter (Topcount, Packard) using a scintillation cocktail (Microscint 0, Packard, Shelton, CT, USA). The results were expressed as a percent inhibition of the control radioligand specific binding. The standard reference compound was OX-A, which was tested in each experiment at several concentrations to obtain a competition curve from which its IC50 was calculated. 

### 2.2. Radioligand Binding Assay on CHO Cells Expressing hOX2R

Cell membrane homogenates (20 µg protein) were incubated for 180 min at 22 °C with 0.04 nM [^125^I] orexin-A in the absence or presence of CBD in a buffer containing 25 mM HEPES/NaOH (pH 7.4), 2.5 mM CaCl_2_, 1 mM MgCl_2_, and 0.5% BSA. Nonspecific binding was determined in the presence of 1 µM OX-B. Following incubation, the samples were filtered rapidly under vacuum through glass fiber filters (GF/B, Packard) presoaked with 0.3% PEI and rinsed several times with ice-cold 50 mM Tris-HCl using a 96-sample cell harvester (Unifilter, Packard). The filters were dried then counted for radioactivity in a scintillation counter (Topcount, Packard) using a scintillation cocktail (Microscint 0, Packard). The results were expressed as a percent inhibition of the control radioligand specific binding. The standard reference compound was OX-B, which was tested in each experiment at several concentrations to obtain a competition curve from which its inhibitory concentration (IC_50_) was calculated.

### 2.3. Cell Culture, Reagents, and Transfections 

Chinese hamster ovary (CHO) cells were propagated in Dulbecco’s modified Eagle’s medium containing 10% FBS, penicillin (100 U/mL), and streptomycin (100 µg/mL) in a humidified atmosphere at 95% O_2_/5% CO_2_ at 37 °C. The day after plating, cells (at a confluence of about 70–80%) were transfected with a plasmid carrying human orexin 1 receptor gene (Origene, IT; cat. n. RG210381). In parallel, a plasmid encoding for a scrambled sequence was used as control (Origene, IT; cat. n. RG) was used as the control condition. To generate stable clones, ORX1-transfected cells were selected using Dulbecco’s modified Eagle’s medium containing Geneticin (G418; 0.6 mg/mL). After one month, the stable overexpression of the human OX1R receptor in CHO cells was measured by quantitative PCR (qPCR). 

### 2.4. Quantitative PCR for Clone Screening

To quantify the OXR1 receptor overexpression in transfected CHO cells and thus select the more efficient clones, the RNA was isolated and purified from both control (not transfected) and OX1R-CHO clones. Quantitative PCR (qPCR) was carried out in a real-time PCR system CFX384 (Bio-Rad, Milan, Italy) using the SsoAdvanced SYBR Green supermix (cat. n. 1725274, Bio-Rad, Milan, Italy) detection technique and specific primers. OX1R forward: CGGAGGAAGACGGCTAAGATG; OX1R reverse: GCTCCCGGAATTTGCCACT; S16 forward: CTGGAGCCTGTTTTGCTTCTG; S16 reverse: TGAGATGGACTGTCGGATGG. Quantitative PCR was performed on separate clones. In addition, each sample was amplified simultaneously in quadruplicate in a one-assay run with a non-template control blank for each primer pair to control for contamination or primer–dimer formation, and the cycle threshold (Ct) value for each experimental group was determined. A housekeeping gene (the ribosomal protein S16) was used to normalize the Ct values, using the 2^−Δ^Ct formula; differences in mRNA content between groups were expressed as 2^−ΔΔ^Ct, as previously described [27].

### 2.5. Calcium Mobilization Assay

CHO cells, wild-type or stably over-expressing recombinant hOX1R, were grown on 100 mm diameter Petri dishes as monolayers as described in Section 2.3. On the day of the experiment, the cells were loaded for 1 h at room temperature with the Ca^2+^ indicator Fluo-4-AM (Thermo-Fisher Scientific, Frosinone Italy) 4 μM in dimethylsulfoxide (DMSO) containing 0.02% Pluronic F-127 (Thermo-Fisher Scientific) in DMEM without FBS. After loading, cells were washed twice in Tyrode’s buffer (145 mM NaCl, 2.5 mM KCl, 1.5 mM CaCl_2_, 1.2 mM MgCl_2_, 10 mM D-glucose, and 10 mM HEPES, pH 7.4) resuspended in the same buffer, and transferred, about 100,000 cells for each determination, to the quartz cuvette of the spectrofluorimeter (λex = 488 nm; λem = 516 nm) Perkin-Elmer LS50B equipped with PTP-1 Fluorescence Peltier System (PerkinElmer Life and Analytical Sciences, Waltham, MA, USA) under continuous stirring at 25 °C. Experiments were conducted by measuring cell fluorescence before and after the addition of the test compound at various concentrations. Antagonist behaviour of CBD was evaluated against the agonist orexin-A (Sigma-Aldrich, Merck, Darmstadt, Germany) 100 nM by adding the test compound in the quartz cuvette 5 min before stimulation of cells with the agonist. The effect on intracellular calcium concentration ([Ca^2+^]_i_), exerted by the agonist alone, was taken as 100%. Data are expressed as the concentration exerting a half-maximal inhibition of agonist-induced [Ca^2+^]_i_ elevation (IC50). Dose-response curves were fitted by a sigmoidal regression with variable slope. Curve fitting and parameter estimation were performed with GraphPad Prism^®^ (GraphPad Software Inc., San Diego, CA, USA). Determinations were performed at least in triplicate. Statistical analysis of the data was performed by analysis of variance at each point using ANOVA followed by Bonferroni’s test. The statistical significance of the antagonist effect was assessed with the Student’s t-test. Statistically significant differences were accepted when the p-value was ≤ 0.05.

### 2.6. Calcium Imaging 

[Ca^2+^]_i_ was measured using the cell-permeable Ca^2+^ indicator, Fura-2AM (Life Technologies), dissolved in DMSO containing 0.02% pluronic F-127 (Life Technologies, Waltham, MA, USA). The transfected cells, previously seeded on polylysine-coated coverslips (300,000 cells/coverslips and 10 independent passages), were loaded with 1 µM Fura-2AM and 1 µM sulfinpyrazone in serum-free medium for 20 min in the dark. The coverslips were placed into a perfusion chamber (Leica Microsystems GmbH, Wetzlar, Germany) mounted on the stage of Leica digital microscope DMI6000 equipped with CO_2_ incubator cage (OkoLab, Burlingame, CA, USA), in order to provide saturated humidity atmosphere containing 95% air and 5% CO_2_ at room temperature. For calcium recording, the cells were incubated in 500 µL of an extracellular solution containing calcium (145 mM NaCl, 2.5 mM KCl, 1.5 mM CaCl_2_, 1.2 mM MgCl_2_, 10 mM D-glucose, and 10 mM HEPES, pH 7.4) and perfused with each drug previously dissolved in the same solution. Observation and acquisition of images were performed with a 20X objective lens and carried out by appropriate speed filter wheels for 387 nm excitation-emission wavelengths (the peak excitation wavelength for calcium-free Fura-2), and 340 nm (the peak excitation wavelength for calcium-bound Fura-2). Images were collected at different times for each drug, to achieve the best setting for every recording: every 5 s for the vehicle and every 1 s for orexin-A, for CBD (both 10 µM and 20 µM), and ionomycin; then the images were digitized and analyzed using LAS AF 2.2.0 Live Data Mode software (Leica Microsystems, Leica, Wetzlar, Germany). An area on each sample free of dye-loaded cells was selected to determine the background fluorescence value to set the baseline value for analysis. The value of emission fluorescence obtained after 340/387 nm excitation was ratiometrically rendered as a measure of the cytosolic Ca^2+^ according to the formula [Ca^2+^]_i_ = KD × (*F*_max_/*F*_min_) × [(*R* − *R*_min_) / (*R*_max_ − *R*)] where KD is the dissociation constant for Fura2 (140 nM) [28], *R* is the experimental emission ratio, and *F*_min_, *R*_max_, *F*_max_, and *R*_min_ are the 380 nm fluorescence emission signals and emission ratio from 340/380 nm excitation at saturating and zero free Ca^2+^ levels, respectively. These values were automatically obtained by applying the software Leica MetaMorph (Leica Microsystems, Leica, Wetzlar, Germany Wetzlar) for calcium imaging.

### 2.7. Computational Methods

Starting ligand geometry was built with Ghemical 2.99.24 [29]. This structure underwent energy minimization (EM) at the molecular mechanics level first, using Tripos 5.2 force field parametrization [30], and then at AM1 semi-empirical level; fully optimized using GAMESS program [31] at the Hartree−Fock level with STO-3G basis set. The resulting conformer was subjected to ab-initio HF/6-31G*/STO-3G single-point calculations to derive the partial atomic charges required by the force field employed in molecular dynamics (MD) simulations (see below) by the Restrained Electrostatic Potential (RESP) procedure [32]. Docking studies were performed with AutoDock 4.2 [33], by using the crystallographic coordinates of OX1R and OX2R (PDB:4ZJC and 4S0V) as starting target structures. Both proteins and ligands were processed with AutoDock Tools (ADT), package version 1.5.6rc17, to merge non-polar hydrogens, calculate Gasteiger charges, and select the rotatable side-chain bonds. Grids for docking evaluation with a spacing of 0.375 Å and 70 × 60 × 70 points, centered on the ligand-binding site (gridcenters −8.224, 0.928, −55.161 and 54.902, 9.686, 53.785 for 4ZJC and 4S0V, respectively), were generated using the program AutoGrid 4.2 included in Autodock 4.2 distribution. OX1R residues, Gln126, Gln179, and Asn318 and the correspondent OX2R Gln134, Gln187, and Asn324, were made flexible during docking runs. The Lamarckian genetic algorithm (LGA) was adopted to perform molecular docking along with the following docking parameters: 100 individuals in a population with a maximum of 15 million energy evaluations and a maximum of 37,000 generations, followed by 300 iterations of Solis and Wets local search. A total of 100 docking runs was performed for each calculation. The loops missing in the crystallographic structures used in this study were modelled with the MODELLER v9.11 program [34]. Representative CBD/OX1R and CBD/OX2R complexes were completed by the addition of all hydrogen atoms and underwent energy minimization. The energy-minimized complexes were embedded in a POPC bilayer using charmmgui web-interface and then molecular dynamics (MD) simulations in membrane environment were carried out with pmemd.cuda module of Amber16 package [35], using lipid 14 (lipids), ff14SB force (protein) [36], and gaff (ligand) [37] force field parameterization. MD production runs were carried out for 100–200 ns. The Cpptraj module of AmberTools16 and program UCSF Chimera 1.10.1 [38] were used to perform MD analysis and to draw the figures, respectively.

## 3. Results and Discussion

The molecular mechanisms responsible for the complex pharmacology of phytocannabinoids in general, including CBD, are still far from being fully understood. Since CBD has received attention for the treatment of drug addiction [12], we considered the orexin receptors as putative molecular targets for this phytocannabinoid. Indeed, biochemical and pharmacological studies [39,40] showed that not only does a cross-talk exist between the endocannabinoid and the hypocretinergic systems, but also that CB_1_R and OX1R receptors form heterodimers, suggesting a synergistic or a mutual modulatory role in common physiopathological functions. In this study, we investigated whether CBD acts as a ligand of orexin receptors.

### 3.1. Radioligand Binding Assay

**Figure 1:**
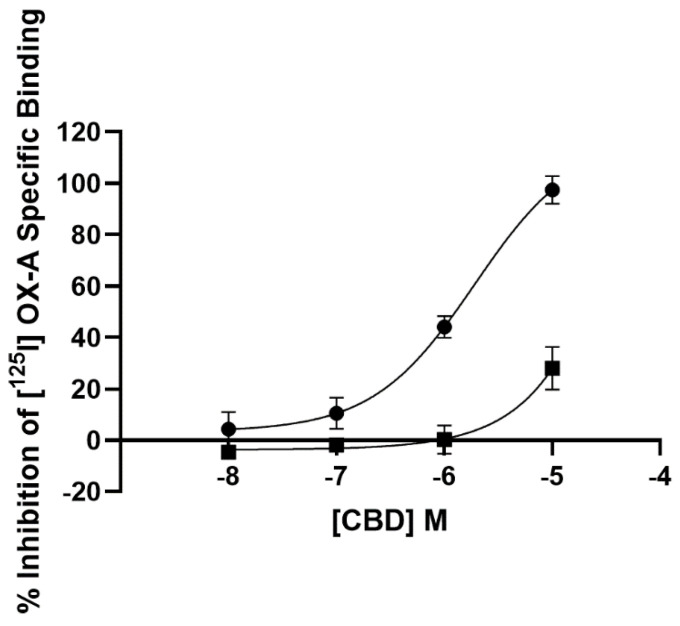
Competition for [^125^I] orexin-A binding to OX1R-CHO cells (filled circle) and OX2R-CHO cells (filled square) by CBD. Data are the mean of three independent experiments; vertical lines show standard deviation.

The affinity of CBD for both OX1R and OX2R was tested on CHO cells overexpressing either receptor. As described by the four-parameter logistic fit (Figure 1), CBD displaced [^125^I] OX-A binding at the OX1R in a concentration-dependent manner with a K_i_ of 1.58 ± 0.2 µM. However, at OX2Rs, CBD was able to only partially (28.0 +/− 8.3%) (*n =* 3) displace such binding at the highest concentration examined (10 µM). Thus, radioligand binding assay showed that CBD selectively binds to OX1R with a K_i_ of 1.58 ± 0.2 µM, a value very close to the clinically achieved concentration (healthy volunteers who received CBD oral solution ~21.4 mg/kg/day for six days achieved a plasma Cmax of 330.3 ng/mL, which equates to 1.04 µM) [41]. 

### 3.2. Calcium Mobilization Assay

**Figure 2:**
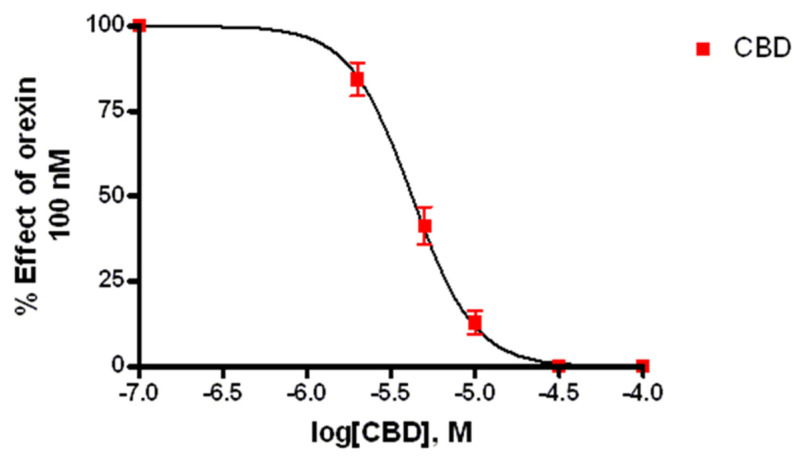
Effect of CBD on Ca^2+^ elevation induced by 100 nM OX-A in CHO cells overexpressing OX1R. Data (mean ± SEM of five independent experiments) are expressed as a percent of the maximal effect observed with OX-A alone (see Figure 3); vertical lines show SEM.

**Figure 3:**
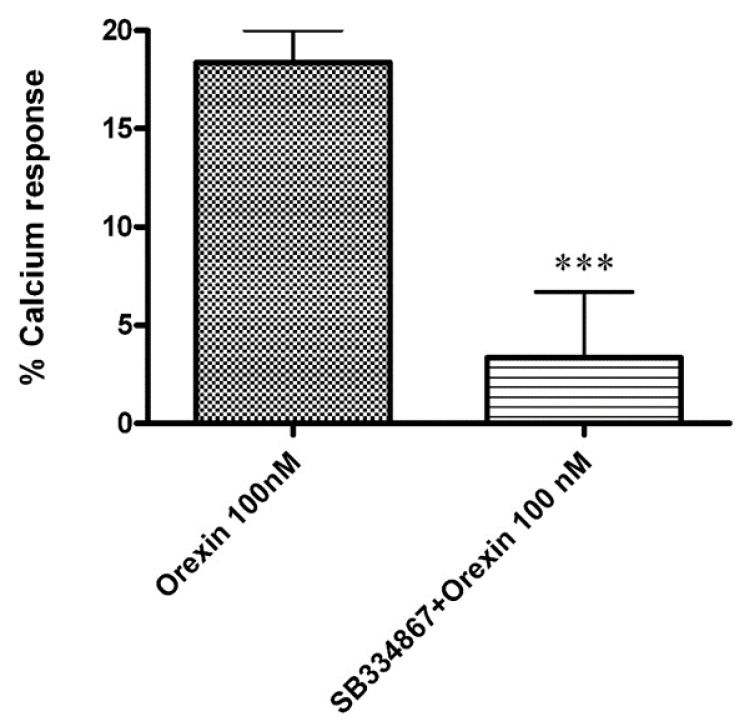
Effect of the OX1R antagonist SB334867 (10 µM) on the Ca^2+^ elevation induced by OX-A (100 nM) in CHO cells overexpressing OX1R. Data are expressed as means ± SEM, (*n* = 4). *** *p* < 0.01.

Ca^2+^ elevations in OX1-CHO cells were measured using the fluorescent Ca^2+^ indicator Fluo-4. Preincubation (5 min) of OX1R-CHO cells with different doses of CBD, followed by incubation with OX-A (100 nM) in 1.5 mM of extracellular Ca^2+^, caused inhibition of the Ca^2+^ elevation due to OX1R response to OX-A. The corresponding dose-inhibition curve is reported in Figure 2 and showed an IC_50_ value of 4.2 ± 0.2 µM (*n =* 5). The OX1R antagonist SB334867 at 10 µM was used as a reference compound to assess the specificity of the antagonist activity of CBD and the inhibition graph is shown in Figure 3. We also tested CBD and OXA in not-transfected CHO cells. In both transfected and not transfected CHO cells CBD caused a slight but significant elevation of intracellular calcium. This effect has been previously reported in several cell types and appears to be independent of direct interaction with Gq [42,43].

### 3.3. Calcium Imaging

#### 3.3.1. OX-A Increases Intracellular [Ca^2+^]_i_ in CHO Cells Stably Expressing OX1R or OX2R

**Figure 4:**
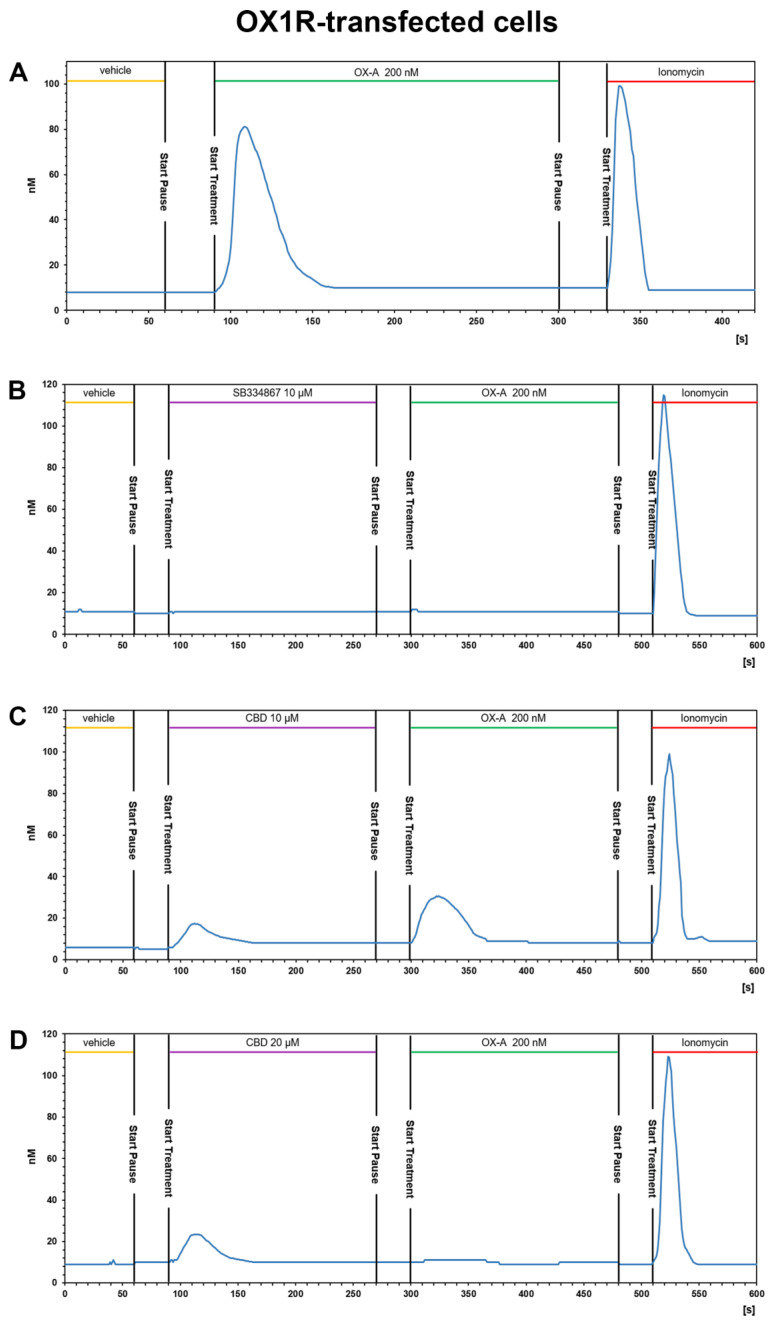
CBD prevents the OX-A-induced [Ca^2+^]_i_ response in CHO cells expressing OX1R. Representative traces of single-cell [Ca^2+^]_i_ response in Fura-2- loaded CHO cells stably expressing OX1R treated with 200 nM OXA, alone or in combination with 10 μM SB334867 or with 10 μM or 20 μM CBD, and finally with ionomycin (2 μM). Images, collected continuously for 420 s (**A**,**B**) or 600 s (**C**,**D**), were analyzed by the Leica MetaMorph software for calcium imaging to quantify the intracellular [Ca^2+^]_i_ increase expressed as 340/387 nm excitation ratio between calcium-bound Fura-2 (green cells) and calcium-free Fura-2 (red cells). (A) 200 nM OX-A increases [Ca^2+^]_i_ in cells. (**B**) The OX1R antagonist SB334867 prevents the OX-A-induced [Ca^2+^]_i_ enhancement in the cells. OX-A-responsive cells became partially (**C**) or completely (**D**) unresponsive to 200 nM OXA after treatment with 10 μM or 20 μM CBD, respectively.

**Figure 5:**
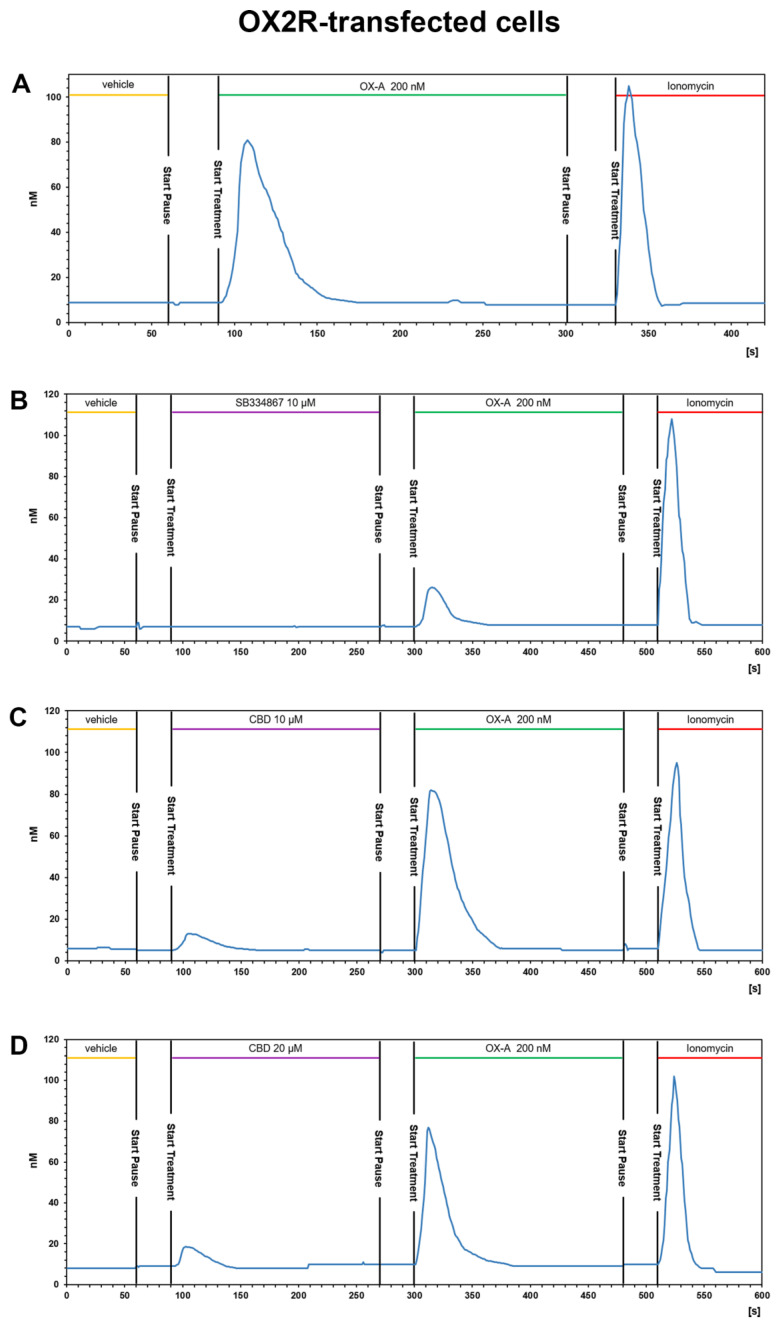
CBD does not alter the OX-A-induced [Ca^2+^]_i_ increase in CHO cells stably transfected with OX2R. Representative traces of single-cell [Ca^2+^]_i_ response in Fura-2- loaded CHO cells stably overexpressing OX2R and treated with 200 nM OXA, alone or in combination with 10μM SB334867 or with 10 μM or 20 μM CBD Ionomycin (2 μM) was added at the end of the experiment. Images, collected continuously for 420 s (**A**,**B**) or 600 s (**C**,**D**), were analyzed by the Leica MetaMorph software for calcium imaging to quantify the intracellular [Ca^2+^]_i_ increase expressed as 340/387nm excitation ratio between calcium-bound Fura-2 (green cells) and calcium-free Fura-2 (red cells). (**A**) 200 nM OX-A increases [Ca^2+^]_i_ in cells. (**B**) The OX1R antagonist SB334867 prevents the OX-A-induced [Ca^2+^]_i_ enhancement in the cells. (**C**,**D**) Treatment with 20 μM or 10 μM CBD, respectively, has no effect on the increase of [Ca^2+^]_i_ induced by OX-A.

OX1R-CHO or OX2R-CHO cells were used for [Ca^2+^]_i_ measurements with fFura-2 fluorescence imaging. OX-A was used at a concentration of 200 nM (chosen according to the effective doses used in our previous study [40]). This concentration of OX-A induced a [Ca^2+^]_i_ increase with a peak of [Ca^2+^]_i_ significantly higher than the basal [Ca^2+^]_i_, both in OX1R-CHO (peak [Ca^2+^]_i_: 81.5 ± 6.1 nM vs. basal [Ca^2+^]_i_: 7.3 ± 1.6 nM; *p* < 0.001) and OX2R-CHO (peak [Ca^2+^]_i_: 80.1 ± 5.7 nM vs. basal [Ca^2+^]_i_: 9.1 ± 1.8 nM; *p* < 0.001) cells. OX-A-induced [Ca^2+^]_i_ increase took place in a fast manner (50–60 s), as shown in a representative graph referred to a randomly recorded single CHO cell stably expressing OX1R (Figure 4A) or OX2R (Figure 5A). The CHO cells stably expressing OX1R did not show [Ca^2+^]_i_ increases in response to 200 nM OXA applied for 180 s after 180 s of treatment with the OX1R antagonist SB334867 (10 μM) in the cell medium (Figure 4B). These results indicate that OX-A directly targets OX1R to regulate [Ca^2+^]_i_ increase in CHO cells stably expressing OX1R. As shown in Figure 5B, and in agreement with its partial antagonistic effect on OX2R, incubation of OX2R-CHO cells with SB334867 10 μM in the calcium-containing buffer solution was able to only partially block the [Ca^2+^]_i_ elevation induced by 200 nM OX-A (peak [Ca^2+^]_i_: 25.7 ± 3.4 nM vs. basal [Ca^2+^]_i_: 7.1 ± 2.6 nM; *p* < 0.05). 

#### 3.3.2. CBD Inhibits the Ca^2+^ Response Induced by OX-A in OX1R, but Not in OX2R Transfected CHO Cells 

The presence of CBD 10 μM or 20 µM for ~3 min in the calcium-containing extracellular solution (see methods section) of CHO cells stably expressing OX1R caused a slight enhancement of [Ca^2+^]_i_ and was able to reduce (10 μM) or prevent (20 μM) the [Ca^2+^]_i_ increase induced by 200 nM OX-A (Figure 4C,D). As shown in Figure 4C, 10 μM CBD induced a slight [Ca^2+^]_i_ peak ([Ca^2+^]_i_: 18.1 ± 2.7 nM vs. basal [Ca^2+^]_i_: 6 ± 1.2 nM; *p* < 0.05) and reduced the increment of [Ca^2+^] induced by subsequent treatment with OX-A ([Ca^2+^]_i_: 30.5 ± 3.6 nM vs. basal [Ca^2+^]_i_: 6 ± 1.2 nM; *p* < 0.05). Pretreatment with 20 μM CBD increased the [Ca^2+^] (peak [Ca^2+^]_i_: 23.7 ± 2.9 nM vs. basal [Ca^2+^]_i_: 8.9 ± 1.3nM; *p* < 0.05), but the [Ca^2+^]_i_ recorded in cells with 200 nM OX-A after CBD treatment was similar to the basal levels (10.8 ± 2.2 nM for OX-A vs. 8.9 ± 1.3 nM for the vehicle-treated cells; *p* > 0.05) (Figure 4D). These results suggest that CBD acts as OX1R antagonist.

**Figure 6:**
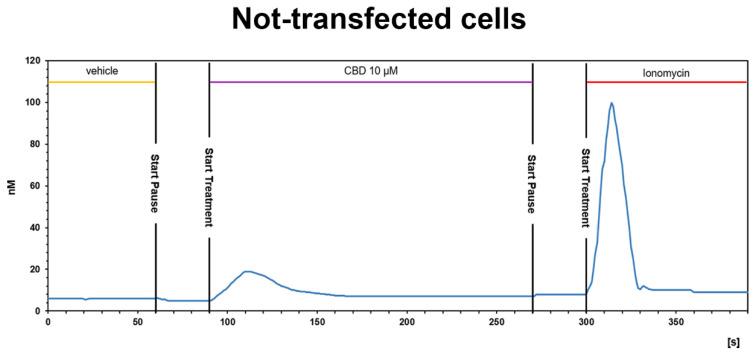
CBD induces a slight [Ca^2+^]_i_ increase in not-transfected CHO cells. Representative traces of single-cell [Ca^2+^]_i_ response in Fura-2- loaded not-transfected CHO cells. Ionomycin (2 μM) was added at the end of the experiment. Images, collected continuously for 390 s, were analyzed by the Leica MetaMorph software for calcium imaging to quantify the intracellular [Ca^2+^]_i_ increase expressed as 340/387 nm excitation ratio between calcium-bound Fura-2 (green cells) and calcium-free Fura-2 (red cells).

On the contrary, pretreatment with 10 μM or 20 µM CBD of CHO cells stably expressing OX2R did not inhibit the Ca^2+^ response induced by 200 nM OX-A. As shown in Figure 5C,D, CBD induced elevation of [Ca^2+^]_i_ in a concentration-dependent manner (12.2 ± 1.3 nM for CBD 10 µM vs. basal level: 5.8 ± 1.6 nM; 17.9 ± 2.3 nM for CBD 20 µM vs. basal level: 7.9 ± 1.9 nM; *p* < 0.05), whereas the addition of OX-A in the same cell medium after 180 s of CBD treatment induced a [Ca^2+^]_i_ enhancement with a peak of [Ca^2+^]_i_ significantly higher than the basal [Ca^2+^]_i_ ([Ca^2+^]_i_: 82.3 ± 5.9 nM after CBD 10µM vs. basal level: 5.8 ± 1.6 nM; 77.5 ± 6.3 nM after CBD 20 µM vs. basal level: 7.9 ± 1.9 nM; *p* < 0.001). The elevation of [Ca^2+^]_i_ induced by CBD, as already observed in calcium mobilization assay, is not due to OXRs, since it also occurs in non-transfected cells (see Figure 6).

Collectively, the functional assays carried out by both intracellular Ca^2+^ measurements and Ca^2+^ imaging showed that CBD is an OX1R antagonist at low micromolar concentrations and is selective for OX1R over OX2R.

### 3.4. OX1R/CBD Theoretical Complex

**Figure 7:**
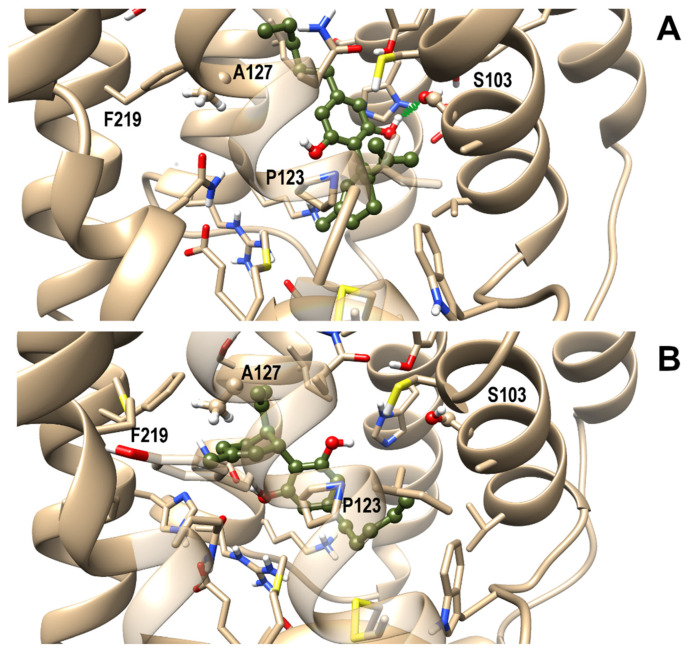
Representative frames from MD of CBD/OX1R complexes: Pose I (**A**) and III (**B**). A ball and stick representation is used for heavy atoms plus polar hydrogens of ligand, whereas a stick representation was used for protein sidechains within 5 Å from the ligand. Protein carbon atoms are colored in tan according to ribbon for protein and olive drab for the ligand. Hydrogen, nitrogen, oxygen, and sulfur atoms are painted white, blue, red and yellow, respectively. A transparent surface for ribbons is used whenever they hide the ligand. A “green spring” style is adopted for H-bonds involving ligand atoms.

Next, a comparative study of molecular docking and molecular dynamics simulations (MD) in membrane environment (up to 200 ns) of CBD in complex with either OX1R or OX2R was undertaken to shed light on the binding mode of CBD in the binding site of OX1R and to rationalize CBD selectivity toward this receptor over OX2R. From docking runs of CBD into the OX1R binding site, four starting poses were selected for the subsequent MD on the basis of binding energy and ability to interact with residues known to be relevant for receptor binding and, among these, three final poses, termed I, II, and III, were stable to MD in terms of root-mean-square-deviation (rmsd). The first two poses shared a similar arrangement, only differing for a slight rotation of the CBD aromatic ring. However, since only pose I formed a stable H-bond with Ser103^2.61^, pose II was discarded from subsequent analysis. In pose I, the terpenoid ring interacted with Pro123^3.29^, a residue involved in ligand interactions also in experimentally determined complex structures, while the pentanoyl chain pointed toward Phe219^5.42^. In pose III CBD adopted a reverse orientation with respect to pose I, with the pentanoyl chain pointing toward the receptor N-terminal end and the terpenoid ring pointing toward Ala127^3.33^, Tyr215^5.38^, and Phe219^5.42^. Pose I and III, representative of the possible binding modes of CBD within the orthosteric binding site of OX1R, are shown in Figure 7, panel a and b, respectively. It is noteworthy that OX1R residues interacting with CBD have been previously characterized as being important for antagonist binding to this receptor [25].

**Figure 8:**
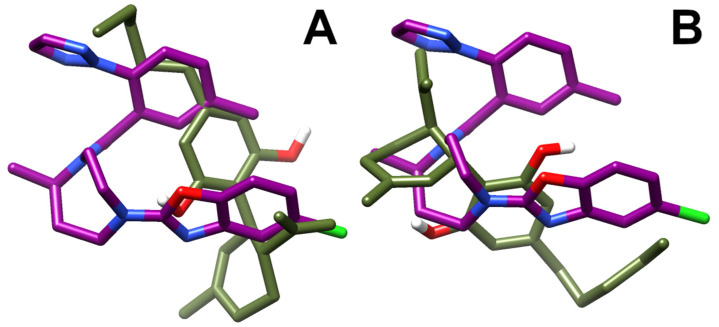
Best-fit superposition of protein backbones between CBD poses I (**A**)/III (**B**) and the x-ray structure of the OX1R-suvorexant complex. Only the ligands are shown for clarity and drawn in stick representation. The suvorexant is colored in deep purple and the CBD in olive drab. Hydrogen, nitrogen, oxygen, and fluorine atoms are painted white, blue, red and green, respectively.

A best-fit superposition of protein backbones between CBD poses I/III and the x-ray structure of the OX1R in complex with the antagonist suvorexant (Figure 8, panels A and B, respectively) shows that, when overlapping the two ligand poses, CBD globally spans the same spatial region occupied by suvorexant, each pose filling about one-half of the volume of the bulkier suvorexant. Hence, the reduced occupancy of the binding site and the higher flexibility of CBD in comparison to suvorexant account for CBD’s lower potency vs. suvorexant (K_i_ 0.55 nM) [44].

**Figure 9:**
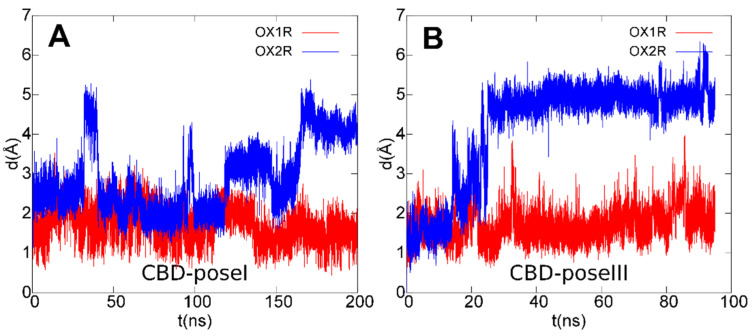
Root mean square deviation (rmsd) plots of CBD ligand in poses I (**A**)/III (**B**) during the last 200 ns (**A**)/95 ns (**B**) of MD simulations after best-fit of protein backbone in OX1R (red) and OX2R (blue).

To assess if the arrangements found for CBD could also be conserved in OX2R, the same starting poses I and III were used for MD of CBD/OX2R complexes. The first pose was also found as the best docking pose in OX2R whereas pose III was not found, due to steric clashes with Thr135^3.33^. Trajectory analysis, reported in Figure 9, showed that neither pose I, nor pose III resulted in stable OX2R, thus explaining the receptor selectivity of CBD toward OX1R. 

In fact, among the contacting residues in the OX1R binding site, CBD formed stable interactions with the only two non-conserved residues between OX1R and OX2R, that is OX1R Ser103^2.61^ (pose I) and OX1R Ala127^3.33^ (pose III), both replaced by a bulkier threonine residue in OX2R, which contributed to the destabilization of the CBD–OX2R complex during MD simulations, in agreement with the results from the binding assays. Furthermore, the docking/MD study identified a protein–ligand interaction network involving OX1R residues reported to be critical for antagonist binding, in agreement with the experimental validation.

## 4. Conclusions

In summary, we identified CBD as a selective OX1R antagonist and such effect could contribute to explaining, for example, the anorexigenic effect exerted by CBD reported in some studies [45], since OX1R is localized in appetite-regulating neurons in the hypothalamus [46] and it has been demonstrated that the hyperphagia induced by the centrally administrated OX-A is mediated by OX1Rs [47,48]. Moreover, the selective OX1R antagonist SB-334867 attenuates orexin-A induced feeding and has anorectic effects inducing satiety without nausea [49]. 

Although further studies are required to assess the clinical relevance of orexin antagonism CBD action, this study adds a new element to the complex picture of the mechanism of action of CBD and paves the way to in vivo studies fully exploring the pharmacological implications of its activity as a negative modulator of the hypocretinergic–endocannabinoid axis, for example in the treatment of substance use disorders or body weight regulation. 

## Data Availability

Not applicable.
